# Chlorido­{2-[(4-chloro­phen­yl)imino­meth­yl]pyridine-κ^2^*N*,*N*′}(η^6^-toluene)­ruthenium(II) hexa­fluoridophosphate

**DOI:** 10.1107/S2414314625001038

**Published:** 2025-03-08

**Authors:** Joel Gichumbi, Holger B. Friedrich, Sizwe J. Zamisa

**Affiliations:** a School of Chemistry and Physics, University of KwaZulu-Natal, Private Bag X54001, Durban, 4000, South Africa; Benemérita Universidad Autónoma de Puebla, México

**Keywords:** crystal structure, piano stool geometry, arene ruthenium compounds, organometallic compound

## Abstract

The asymmetric unit of the title compound, [RuCl(C_7_H_8_)(C_12_H_9_ClN_2_)]PF_6_, contains two cationic ruthenium(II) complexes and two [PF_6_]^−^ anions. One of the anions exhibits disorder over two positions. The ruthenium complex adopts a piano-stool geometry, with chelating Schiff base and chlorido ligands occupying three coordination sites, and the arene ring serving as the ‘seat’.

## Abstract 2

The crystal structure of an arene ruthenium(II) complex bearing a bidentate Schiff base ligand is reported.

## Graphical abstract

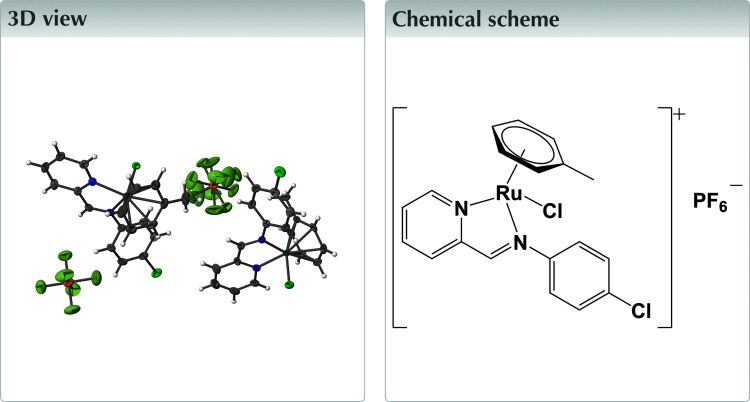

## Structure description

Arene ruthenium compounds belong to a family of robust metal–organic mol­ecules that played an important role in the development of organometallic chemistry (Gichumbi & Friedrich, 2018 ▸). There has been an intense research inter­est in the chemistry of these arene complexes with mono-, di-, or poly-dentate ligands. The arene precursor complex undergoes cleavage of the chloride bridges with various two-electron donor ligands to give mononuclear complexes, while reactions with bidentate ligands afford cationic complexes (Gichumbi *et al.*, 2016*a* ▸, 2020 ▸, 2021 ▸).

**Figure 1:**
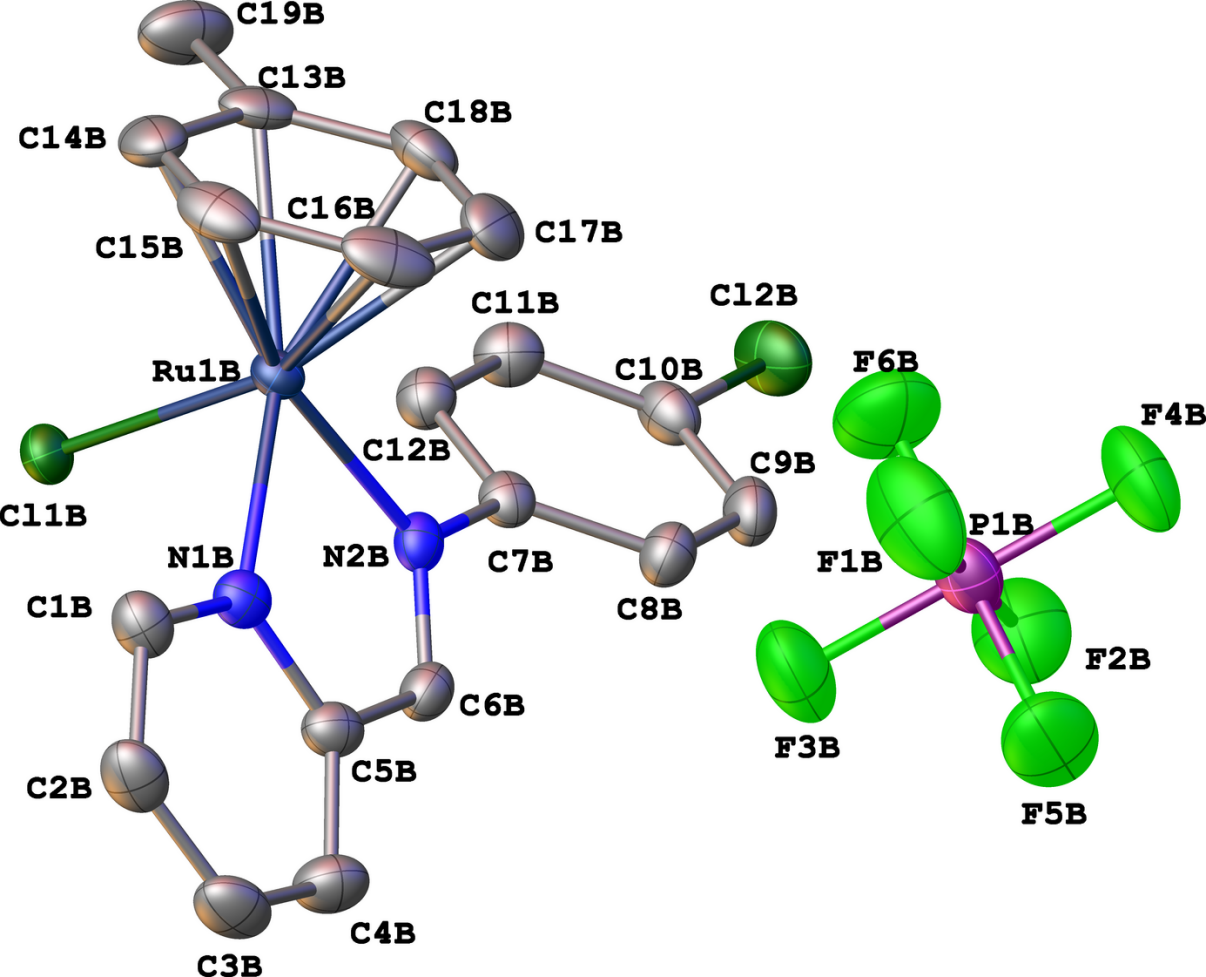
The structures of the molecular entities in the crystal of the title compound with displacement ellipsoids drawn at the 50% probability level. One of the two mol­ecular components of the title compound and all hydrogen atoms have been omitted for clarity.

**Figure 2:**
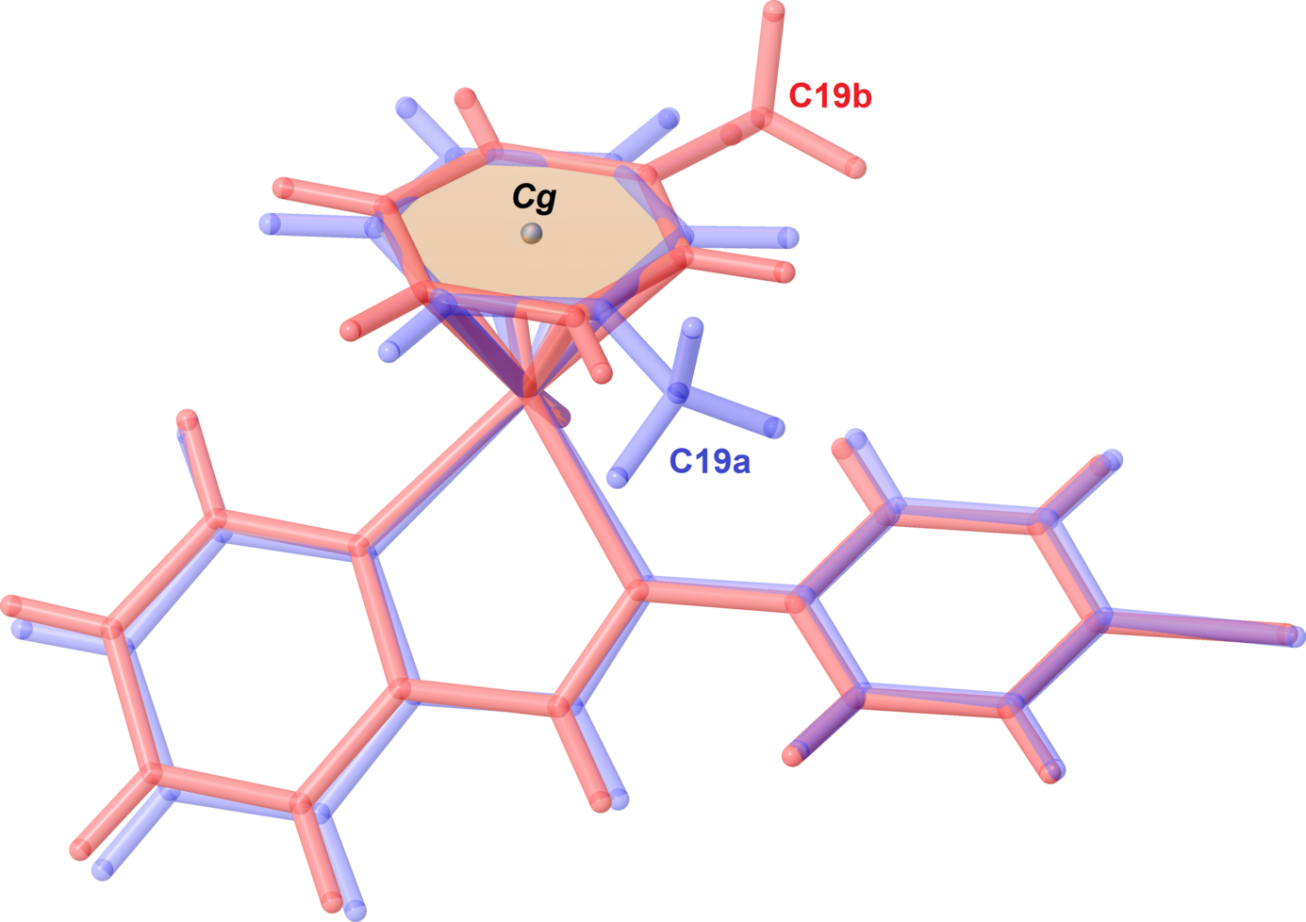
Mol­ecular overlay diagram of the two cationic Ru^II^ complexes in the asymmetric unit of the title compound. The blue- and red-coloured cationic species correspond to those of mol­ecules *A* and *B*, respectively.

**Figure 3:**
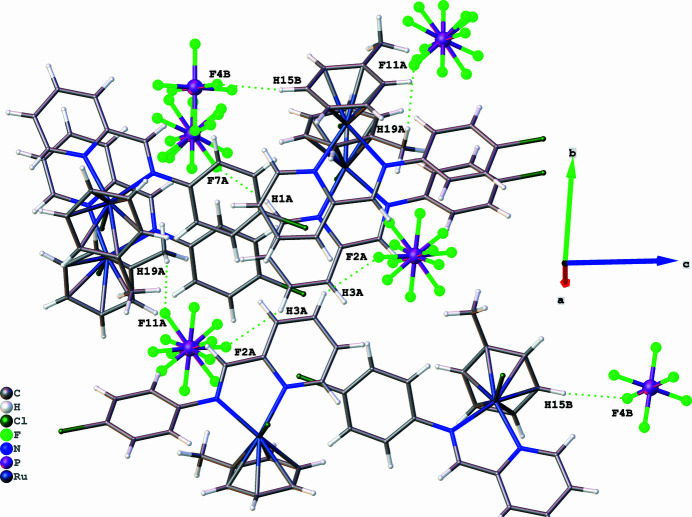
Representation of inter­molecular C—H⋯F hydrogen-bonding patterns (green dotted bonds) in the crystal packing of the title compound.

The asymmetric unit of the title compound contains two cationic ruthenium complexes and two [PF_6_]^−^ anions. Each cationic ruthenium(II) complex shows a piano-stool geometry, where the chelating ligand and the chloride atom occupy the positions of three legs of a piano stool, and the arene ring occupies the remaining coordination sites as the seat of the stool (Fig. 1 ▸). The Ru—N and Ru—Cl bond lengths were found to be 2.081 (3)–2.090 (2) Å and 2.3764 (8)–2.3821 (8) Å, respectively. Furthermore, the N—Ru—N and N—Ru—Cl bond angles range from 76.82 (10) to 76.84 (10) and from 85.76 (7) to 86.85 (7)°, respectively. These bond parameters are comparable to those reported for other arene ruthenium complexes with *N*,*N*′-donor ligands (Gichumbi *et al.*, 2016*b* ▸, 2017 ▸, 2018 ▸; Gichumbi & Friedrich, 2018 ▸; Zamisa *et al.*, 2024 ▸). A mol­ecular overlay diagram of the two cationic species in the asymmetric unit of the title compound reveals significant geometric differences of the η^6^-toluene ligand (Fig. 2 ▸) with a root-mean-square deviation (RMSD) value of 1.358 Å. Furthermore, the arene rings of the two cationic species appear to be rotated by 96.97 (16)–99.13 (15)° with respect to each other when considering either an C19*a*⋯*Cg*(mol­ecule *A* arene)⋯C19*b* or C19*b*⋯*Cg*(mol­ecule *B* arene)⋯C19*a* angle. The crystal packing of the title compound is stabilized by various inter­molecular C—H⋯F hydrogen bonds between aromatic or methyl hydrogen atoms of the cationic ruthenium(II) complex and the fluorine atoms of the [PF_6_]^−^ anions (Table 1 ▸, Fig. 3 ▸).

## Synthesis and crystallization

To a suspension of [(η^6^-toluene)Ru(μ-Cl)Cl]_2_ (0.2 mmol) in aceto­nitrile (20 ml) was added the pyridine-imine ligand (0.42 mmol). The mixture was stirred at room temperature for 3 h followed by reduction in the volume of the solvent *in vacuo* to about 10 ml before adding NH_4_PF_6_ (0.42 mmol). The mixture was then cooled in an ice bath while stirring for 2 h leading to a precipitate, which was collected by filtration. The filtrate was washed with diethyl ether and dried *in vacuo*. Crystals suitable for single-crystal X-ray diffraction studies were grown by layering solutions of the compound in acetone with hexane and leaving undisturbed for 2 d.

Red solid, yield 82%, m.p. 200°C (decomp.). ^1^H NMR (400 MHz, DMSO-*d*_6_): δ_p.p.m._ 9.61 (*d*, *J*_HH_ = 5.4 Hz, 1H, py); 8.92 (*s*,1*H*, CH=N); 8.31 (*m*, 2H, py); 7.90 (*m*, 1H, py); 7.83 (*m*, 2H, Ph); 7.72 (*d*, *J*_HH_ = 8.72 Hz, 2H, Ph); 6.16 (*m*,1*H*, Arene); 5.83 (*m*, 1H, Arene); 5.78 (*m*, 2H, Arene); 5.59 (*d*, *J*_HH_ = 3.68 Hz, 1H, Arene); 2.12 (*s*, 3H, Arene). ^13^C NMR (100 MHz, DMSO-*d*_6_): δ_p.p.m._ 168.47 (py), 156.13 (py), 154.55 (py), 150.37 (py), 139.92 (py), 133.92 (py), 130.03 (Ar), 129.46 (Ar), 128.88 (Ar), 124.30 (Ar), 105.30 (Ar), 90.62 (Ar), 90.09 (Ar), 84.13 (Ar), 84.02 (Ar), 80.91 (Ar), 18.58 (Me). MS (ESI+, *m*/*z*): 444.98 [C_19_H_17_Cl_2_N_2_Ru]^+^.

## Refinement

Crystallographic data and structure refinement details are summarized in Table 2 ▸. The fluorine atoms of one PF_6_^−^ anion were found to be disordered over two positions. PART 1 and 2 instructions were used to model the disorder and the major component was refined with a site occupancy of 0.583 (6). Furthermore, the refinement of the disordered PF_6_^−^ species was kept stable using standard parameters of SADI (same distances) and DELU (rigid bond) restraints. The fluorine atoms were further restrained using SIMU with a standard deviation of 0.02 Å^2^ to have the same displacement components (Sheldrick, 2015 ▸).

## Supplementary Material

Crystal structure: contains datablock(s) I. DOI: 10.1107/S2414314625001038/bh4091sup1.cif

Structure factors: contains datablock(s) I. DOI: 10.1107/S2414314625001038/bh4091Isup2.hkl

CCDC reference: 2421509

Additional supporting information:  crystallographic information; 3D view; checkCIF report

## Figures and Tables

**Table 1:** Hydrogen-bond geometry (Å, °)

*D*—H⋯*A*	*D*—H	H⋯*A*	*D*⋯*A*	*D*—H⋯*A*
C1*A*—H1*A*⋯F7*A*^i^	0.95	2.33	3.242 (11)	160
C3*A*—H3*A*⋯F2*A*^ii^	0.95	2.43	3.261 (8)	147
C19*A*—H19*A*⋯F11*A*^iii^	0.98	2.38	3.243 (9)	146
C15*B*—H15*B*⋯F4*B*^iv^	0.95	2.35	3.292 (4)	172

Symmetry codes: (i) 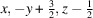; (ii) 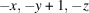; (iii) 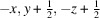; (iv) 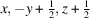.

**Table 2:** Experimental details

Crystal data	
Chemical formula	[RuCl(C_7_H_8_)(C_12_H_9_ClN_2_)]PF_6_
*M*_r_	590.28
Crystal system, space group	Monoclinic, *P*2_1_/*c*
Temperature (K)	173
*a*, *b*, *c* (Å)	15.4238 (13), 16.1528 (14), 18.2354 (16)
β (°)	111.508 (2)
*V* (Å^3^)	4226.8 (6)
*Z*	8
Radiation type	Mo *K*α
μ (mm^−1^)	1.13
Crystal size (mm)	0.14 × 0.11 × 0.09

Data collection	
Diffractometer	Bruker Kappa Duo APEXII
Absorption correction	Multi-scan (*SADABS*; Krause *et al.*, 2015 ▸)
*T*_min_, *T*_max_	0.920, 1.000
No. of measured, independent and observed [*I* > 2σ(*I*)] reflections	74571, 10189, 7517
*R*_int_	0.080
(sin θ/λ)_max_ (Å^−1^)	0.661

Refinement	
*R*[*F*^2^ > 2σ(*F*^2^)], *wR*(*F*^2^), *S*	0.038, 0.089, 1.03
No. of reflections	10189
No. of parameters	616
No. of restraints	157
H-atom treatment	H-atom parameters constrained
Δρ_max_, Δρ_min_ (e Å^−3^)	0.75, −0.64

Computer programs: *APEX2* and *SAINT* (Bruker, 2010 ▸), *SHELXS2013* (Sheldrick, 2008 ▸), *SHELXL2018/3* (Sheldrick, 2015 ▸) and *OLEX2* (Dolomanov *et al.*, 2009 ▸).

## full crystallographic data

### Chlorido{2-[(4-chlorophenyl)iminomethyl]pyridine-κ2N,N'}(η6-toluene)ruthenium(II) hexafluoridophosphate Crystal data

**Table:**

[RuCl(C_7_H_8_)(C_12_H_9_ClN_2_)]PF_6_	*F*(000) = 2336
*M**_r_* = 590.28	*D*_x_ = 1.855 Mg m^−^^3^
Monoclinic, *P*2_1_/*c*	Mo *K*α radiation, λ = 0.71073 Å
*a* = 15.4238 (13) Å	Cell parameters from 5713 reflections
*b* = 16.1528 (14) Å	θ = 2.4–27.8°
*c* = 18.2354 (16) Å	µ = 1.13 mm^−^^1^
β = 111.508 (2)°	*T* = 173 K
*V* = 4226.8 (6) Å^3^	Block, red
*Z* = 8	0.14 × 0.11 × 0.09 mm

### Chlorido{2-[(4-chlorophenyl)iminomethyl]pyridine-κ2N,N'}(η6-toluene)ruthenium(II) hexafluoridophosphate Data collection

**Table:**

Bruker Kappa Duo APEXII Diffractometer	10189 independent reflections
Radiation source: fine-focus sealed tube, Incoatec Iµs	7517 reflections with *I* > 2σ(*I*)
Mirror optics monochromator	*R*_int_ = 0.080
Detector resolution: 7.9 pixels mm^-1^	θ_max_ = 28.0°, θ_min_ = 1.4°
ω and φ scans	*h* = −20→20
Absorption correction: multi-scan (SADABS; Krause *et al.*, 2015)	*k* = −21→21
*T*_min_ = 0.920, *T*_max_ = 1.000	*l* = −24→24
74571 measured reflections	

### Chlorido{2-[(4-chlorophenyl)iminomethyl]pyridine-κ2N,N'}(η6-toluene)ruthenium(II) hexafluoridophosphate Refinement

**Table:**

Refinement on *F*^2^	Primary atom site location: structure-invariant direct methods
Least-squares matrix: full	Secondary atom site location: difference Fourier map
*R*[*F*^2^ > 2σ(*F*^2^)] = 0.038	Hydrogen site location: inferred from neighbouring sites
*wR*(*F*^2^) = 0.089	H-atom parameters constrained
*S* = 1.03	*w* = 1/[σ^2^(*F*_o_^2^) + (0.0305*P*)^2^ + 2.789*P*] where *P* = (*F*_o_^2^ + 2*F*_c_^2^)/3
10189 reflections	(Δ/σ)_max_ = 0.001
616 parameters	Δρ_max_ = 0.75 e Å^−^^3^
157 restraints	Δρ_min_ = −0.64 e Å^−^^3^

### Chlorido{2-[(4-chlorophenyl)iminomethyl]pyridine-κ2N,N'}(η6-toluene)ruthenium(II) hexafluoridophosphate Fractional atomic coordinates and isotropic or equivalent isotropic displacement parameters (Å^2^)

**Table:**

	*x*	*y*	*z*	*U*_iso_*/*U*_eq_	Occ. (<1)
F7A	0.0628 (9)	0.6670 (7)	0.2924 (7)	0.111 (4)	0.417 (6)
F9A	−0.0682 (9)	0.5980 (9)	0.2756 (8)	0.104 (4)	0.417 (6)
F10A	0.0691 (11)	0.6026 (9)	0.1885 (10)	0.121 (4)	0.417 (6)
Ru1A	0.16363 (2)	0.84822 (2)	0.06950 (2)	0.01747 (7)	
Cl1A	0.30832 (5)	0.85330 (5)	0.05113 (5)	0.02794 (18)	
Cl2A	0.42931 (7)	0.88497 (6)	0.49474 (5)	0.0417 (2)	
P1A	0.00425 (6)	0.60271 (6)	0.23620 (5)	0.0278 (2)	
F1A	0.0904 (4)	0.6283 (5)	0.3134 (3)	0.069 (2)	0.583 (6)
F2A	−0.0806 (3)	0.5824 (5)	0.1594 (3)	0.080 (2)	0.583 (6)
F3A	−0.0482 (5)	0.5694 (4)	0.2904 (4)	0.0562 (19)	0.583 (6)
F4A	0.0639 (6)	0.6429 (4)	0.1896 (6)	0.0697 (19)	0.583 (6)
F5A	0.0485 (4)	0.5176 (3)	0.2322 (5)	0.0749 (19)	0.583 (6)
F6A	−0.0379 (4)	0.6917 (3)	0.2413 (5)	0.0744 (19)	0.583 (6)
F8A	−0.0559 (9)	0.5347 (6)	0.1813 (6)	0.147 (5)	0.417 (6)
F11A	0.0598 (5)	0.5324 (5)	0.2940 (6)	0.085 (3)	0.417 (6)
F12A	−0.0563 (5)	0.6677 (5)	0.1767 (5)	0.066 (2)	0.417 (6)
N1A	0.14963 (17)	0.73032 (16)	0.02050 (15)	0.0226 (6)	
N2A	0.23222 (17)	0.77288 (16)	0.16674 (14)	0.0201 (5)	
C1A	0.1114 (2)	0.7115 (2)	−0.05628 (19)	0.0270 (7)	
H1A	0.089573	0.754995	−0.093628	0.032*	
C2A	0.1028 (2)	0.6307 (2)	−0.0826 (2)	0.0347 (9)	
H2A	0.075011	0.619271	−0.137443	0.042*	
C3A	0.1343 (3)	0.5667 (2)	−0.0299 (2)	0.0391 (9)	
H3A	0.127392	0.510838	−0.047319	0.047*	
C4A	0.1766 (2)	0.5858 (2)	0.0495 (2)	0.0359 (9)	
H4A	0.199729	0.543025	0.087521	0.043*	
C5A	0.1845 (2)	0.6677 (2)	0.07264 (19)	0.0249 (7)	
C6A	0.2307 (2)	0.6952 (2)	0.15299 (18)	0.0237 (7)	
H6A	0.259010	0.656724	0.194301	0.028*	
C7A	0.2788 (2)	0.80110 (19)	0.24635 (17)	0.0214 (7)	
C8A	0.3353 (2)	0.8706 (2)	0.26042 (19)	0.0259 (7)	
H8A	0.342276	0.899942	0.217768	0.031*	
C9A	0.3813 (2)	0.8969 (2)	0.33707 (19)	0.0278 (8)	
H9A	0.420825	0.944006	0.347691	0.033*	
C10A	0.3691 (2)	0.8539 (2)	0.39808 (19)	0.0290 (8)	
C11A	0.3109 (2)	0.7859 (2)	0.38443 (18)	0.0274 (7)	
H11A	0.302389	0.757847	0.427085	0.033*	
C12A	0.2653 (2)	0.7594 (2)	0.30787 (18)	0.0255 (7)	
H12A	0.224835	0.712896	0.297361	0.031*	
C13A	0.0670 (2)	0.8963 (2)	0.12295 (19)	0.0247 (7)	
C14A	0.1374 (2)	0.9567 (2)	0.1308 (2)	0.0284 (8)	
H14A	0.172032	0.978788	0.181375	0.034*	
C15A	0.1563 (2)	0.9837 (2)	0.0660 (2)	0.0339 (8)	
H15A	0.204438	1.022983	0.072647	0.041*	
C16A	0.1036 (2)	0.9527 (2)	−0.0104 (2)	0.0335 (9)	
H16A	0.115605	0.971601	−0.055072	0.040*	
C17A	0.0344 (2)	0.8944 (2)	−0.01880 (19)	0.0307 (8)	
H17A	−0.001061	0.873571	−0.069751	0.037*	
C18A	0.0157 (2)	0.8654 (2)	0.0470 (2)	0.0267 (7)	
H18A	−0.031447	0.825121	0.040154	0.032*	
C19A	0.0494 (3)	0.8646 (3)	0.1933 (2)	0.0419 (10)	
H19A	−0.000030	0.897490	0.201044	0.063*	
H19B	0.106536	0.869097	0.240177	0.063*	
H19C	0.029962	0.806506	0.184859	0.063*	
Ru1B	0.34307 (2)	0.33543 (2)	0.42690 (2)	0.01810 (7)	
Cl1B	0.19856 (5)	0.36287 (5)	0.44160 (5)	0.02399 (17)	
Cl2B	0.08970 (7)	0.34750 (6)	0.00329 (5)	0.0395 (2)	
P1B	0.49695 (7)	0.10602 (7)	0.25810 (5)	0.0351 (2)	
F1B	0.58410 (18)	0.1183 (2)	0.33663 (13)	0.0728 (9)	
F2B	0.40965 (16)	0.09432 (18)	0.17865 (14)	0.0640 (7)	
F3B	0.43110 (17)	0.09712 (17)	0.30747 (15)	0.0632 (7)	
F4B	0.56327 (16)	0.11489 (18)	0.20892 (13)	0.0589 (7)	
F5B	0.5138 (2)	0.00892 (16)	0.26382 (17)	0.0713 (8)	
F6B	0.4779 (2)	0.20238 (16)	0.25130 (17)	0.0750 (8)	
N1B	0.34625 (17)	0.22625 (16)	0.48847 (15)	0.0223 (6)	
N2B	0.27009 (17)	0.25245 (16)	0.33833 (14)	0.0210 (6)	
C1B	0.3824 (2)	0.2166 (2)	0.56678 (18)	0.0272 (7)	
H1B	0.406219	0.263764	0.599037	0.033*	
C2B	0.3863 (2)	0.1406 (2)	0.6023 (2)	0.0322 (8)	
H2B	0.412190	0.135867	0.658062	0.039*	
C3B	0.3524 (2)	0.0713 (2)	0.5561 (2)	0.0350 (9)	
H3B	0.357138	0.018036	0.579430	0.042*	
C4B	0.3113 (2)	0.0812 (2)	0.4752 (2)	0.0334 (8)	
H4B	0.285096	0.035139	0.442132	0.040*	
C5B	0.3090 (2)	0.1591 (2)	0.44323 (19)	0.0244 (7)	
C6B	0.2658 (2)	0.1777 (2)	0.35988 (19)	0.0242 (7)	
H6B	0.235425	0.135931	0.322651	0.029*	
C7B	0.2267 (2)	0.2743 (2)	0.25661 (17)	0.0219 (7)	
C8B	0.2354 (2)	0.2238 (2)	0.19851 (18)	0.0271 (7)	
H8B	0.270402	0.174009	0.212203	0.033*	
C9B	0.1927 (2)	0.2464 (2)	0.12023 (19)	0.0302 (8)	
H9B	0.197795	0.212221	0.079599	0.036*	
C10B	0.1430 (2)	0.3191 (2)	0.10210 (18)	0.0279 (7)	
C11B	0.1348 (2)	0.3710 (2)	0.15957 (19)	0.0289 (8)	
H11B	0.100670	0.421212	0.145506	0.035*	
C12B	0.1772 (2)	0.3484 (2)	0.23805 (19)	0.0258 (7)	
H12B	0.172600	0.382963	0.278597	0.031*	
C13B	0.3619 (2)	0.4668 (2)	0.3996 (2)	0.0283 (8)	
C14B	0.3997 (2)	0.4533 (2)	0.4831 (2)	0.0320 (8)	
H14B	0.379411	0.486742	0.516654	0.038*	
C15B	0.4657 (2)	0.3918 (2)	0.5154 (2)	0.0336 (9)	
H15B	0.492026	0.384447	0.570915	0.040*	
C16B	0.4935 (2)	0.3403 (2)	0.4658 (2)	0.0378 (9)	
H16B	0.537474	0.297362	0.487839	0.045*	
C17B	0.4568 (2)	0.3520 (2)	0.3840 (2)	0.0350 (9)	
H17B	0.475427	0.317339	0.350349	0.042*	
C18B	0.3915 (2)	0.4163 (2)	0.3524 (2)	0.0314 (8)	
H18B	0.367249	0.425007	0.297038	0.038*	
C19B	0.2906 (3)	0.5326 (2)	0.3657 (3)	0.0455 (10)	
H19D	0.321157	0.586785	0.373632	0.068*	
H19E	0.245434	0.531547	0.392066	0.068*	
H19F	0.258181	0.522634	0.309193	0.068*	

### Chlorido{2-[(4-chlorophenyl)iminomethyl]pyridine-κ2N,N'}(η6-toluene)ruthenium(II) hexafluoridophosphate Atomic displacement parameters (Å^2^)

**Table:**

	*U*^11^	*U*^22^	*U*^33^	*U*^12^	*U*^13^	*U*^23^
F7A	0.128 (8)	0.076 (7)	0.089 (7)	−0.052 (6)	−0.006 (6)	−0.014 (5)
F9A	0.086 (6)	0.160 (10)	0.091 (6)	0.053 (6)	0.064 (5)	0.082 (6)
F10A	0.128 (7)	0.162 (11)	0.125 (7)	0.027 (9)	0.109 (5)	0.015 (9)
Ru1A	0.01686 (12)	0.01832 (13)	0.01865 (13)	0.00269 (10)	0.00819 (10)	0.00200 (9)
Cl1A	0.0213 (4)	0.0327 (5)	0.0353 (4)	0.0004 (3)	0.0168 (3)	0.0016 (3)
Cl2A	0.0528 (6)	0.0424 (6)	0.0227 (4)	0.0067 (4)	0.0054 (4)	−0.0047 (4)
P1A	0.0312 (5)	0.0294 (5)	0.0227 (4)	−0.0008 (4)	0.0097 (4)	−0.0003 (4)
F1A	0.036 (3)	0.110 (6)	0.049 (3)	−0.008 (3)	0.002 (2)	−0.016 (3)
F2A	0.049 (3)	0.142 (7)	0.034 (3)	−0.006 (4)	−0.003 (2)	−0.016 (3)
F3A	0.073 (4)	0.060 (4)	0.053 (4)	−0.021 (3)	0.043 (3)	0.006 (3)
F4A	0.087 (4)	0.057 (4)	0.097 (4)	−0.024 (4)	0.071 (3)	−0.003 (4)
F5A	0.087 (4)	0.030 (3)	0.136 (5)	0.011 (2)	0.074 (4)	−0.006 (3)
F6A	0.073 (3)	0.037 (3)	0.132 (5)	0.013 (2)	0.059 (4)	0.000 (3)
F8A	0.200 (10)	0.059 (6)	0.084 (6)	−0.030 (6)	−0.063 (6)	−0.015 (5)
F11A	0.064 (4)	0.074 (5)	0.091 (6)	0.019 (4)	−0.002 (4)	0.047 (5)
F12A	0.062 (4)	0.065 (5)	0.071 (5)	0.016 (4)	0.025 (4)	0.044 (4)
N1A	0.0193 (13)	0.0256 (15)	0.0245 (14)	0.0011 (11)	0.0101 (11)	−0.0012 (11)
N2A	0.0189 (13)	0.0205 (14)	0.0221 (13)	0.0011 (10)	0.0091 (10)	0.0025 (10)
C1A	0.0233 (17)	0.033 (2)	0.0277 (17)	−0.0008 (14)	0.0127 (14)	−0.0047 (14)
C2A	0.0335 (19)	0.041 (2)	0.033 (2)	−0.0049 (16)	0.0171 (16)	−0.0130 (16)
C3A	0.042 (2)	0.029 (2)	0.051 (2)	−0.0045 (17)	0.0219 (19)	−0.0137 (18)
C4A	0.039 (2)	0.026 (2)	0.043 (2)	0.0043 (16)	0.0159 (17)	−0.0030 (16)
C5A	0.0262 (16)	0.0197 (17)	0.0306 (18)	0.0037 (13)	0.0126 (14)	−0.0011 (13)
C6A	0.0220 (16)	0.0231 (18)	0.0256 (16)	0.0033 (13)	0.0082 (13)	0.0050 (13)
C7A	0.0189 (15)	0.0229 (17)	0.0195 (15)	0.0040 (12)	0.0038 (12)	0.0025 (12)
C8A	0.0268 (17)	0.0269 (18)	0.0244 (17)	0.0013 (14)	0.0099 (13)	0.0053 (13)
C9A	0.0252 (17)	0.0258 (19)	0.0279 (17)	−0.0018 (14)	0.0043 (14)	0.0008 (14)
C10A	0.0298 (18)	0.033 (2)	0.0202 (16)	0.0092 (15)	0.0049 (14)	0.0003 (14)
C11A	0.0292 (18)	0.031 (2)	0.0227 (17)	0.0065 (15)	0.0106 (14)	0.0072 (14)
C12A	0.0207 (16)	0.0265 (18)	0.0290 (18)	0.0033 (13)	0.0087 (14)	0.0091 (14)
C13A	0.0249 (16)	0.0234 (18)	0.0314 (18)	0.0127 (13)	0.0168 (14)	0.0064 (14)
C14A	0.0331 (18)	0.0210 (18)	0.0320 (18)	0.0106 (14)	0.0129 (15)	−0.0032 (14)
C15A	0.0315 (19)	0.0193 (18)	0.057 (2)	0.0060 (14)	0.0238 (18)	0.0085 (16)
C16A	0.0354 (19)	0.037 (2)	0.036 (2)	0.0184 (16)	0.0221 (16)	0.0180 (16)
C17A	0.0255 (17)	0.040 (2)	0.0224 (17)	0.0134 (15)	0.0042 (14)	0.0037 (15)
C18A	0.0188 (15)	0.0252 (18)	0.0382 (19)	0.0061 (13)	0.0131 (14)	−0.0006 (14)
C19A	0.050 (2)	0.048 (3)	0.042 (2)	0.0196 (19)	0.0338 (19)	0.0145 (18)
Ru1B	0.01450 (12)	0.02020 (14)	0.01910 (13)	−0.00135 (10)	0.00555 (9)	0.00131 (10)
Cl1B	0.0208 (4)	0.0265 (4)	0.0280 (4)	0.0007 (3)	0.0129 (3)	−0.0008 (3)
Cl2B	0.0491 (5)	0.0455 (6)	0.0210 (4)	−0.0067 (4)	0.0095 (4)	0.0034 (4)
P1B	0.0382 (5)	0.0401 (6)	0.0285 (5)	0.0053 (4)	0.0139 (4)	0.0087 (4)
F1B	0.0568 (16)	0.123 (3)	0.0316 (13)	−0.0045 (16)	0.0079 (12)	0.0079 (14)
F2B	0.0480 (15)	0.084 (2)	0.0505 (15)	−0.0027 (14)	0.0065 (12)	−0.0033 (14)
F3B	0.0657 (17)	0.075 (2)	0.0670 (17)	0.0116 (14)	0.0456 (14)	0.0145 (14)
F4B	0.0522 (15)	0.094 (2)	0.0388 (13)	−0.0044 (14)	0.0264 (11)	0.0054 (13)
F5B	0.089 (2)	0.0458 (16)	0.094 (2)	0.0224 (14)	0.0500 (17)	0.0178 (14)
F6B	0.107 (2)	0.0399 (16)	0.0815 (19)	0.0105 (15)	0.0385 (17)	0.0116 (14)
N1B	0.0177 (13)	0.0241 (15)	0.0248 (14)	0.0023 (11)	0.0073 (11)	0.0021 (11)
N2B	0.0170 (13)	0.0255 (15)	0.0214 (13)	0.0008 (11)	0.0080 (10)	−0.0028 (11)
C1B	0.0247 (17)	0.032 (2)	0.0254 (17)	0.0039 (14)	0.0096 (14)	0.0047 (14)
C2B	0.0344 (19)	0.035 (2)	0.0295 (18)	0.0101 (16)	0.0139 (15)	0.0133 (15)
C3B	0.036 (2)	0.027 (2)	0.046 (2)	0.0075 (16)	0.0194 (17)	0.0152 (16)
C4B	0.035 (2)	0.0229 (19)	0.041 (2)	−0.0004 (15)	0.0124 (16)	0.0028 (15)
C5B	0.0207 (15)	0.0223 (18)	0.0307 (18)	0.0007 (13)	0.0099 (13)	0.0020 (13)
C6B	0.0229 (16)	0.0231 (18)	0.0274 (17)	−0.0007 (13)	0.0102 (13)	−0.0055 (13)
C7B	0.0175 (15)	0.0257 (18)	0.0219 (16)	−0.0027 (13)	0.0063 (12)	−0.0017 (13)
C8B	0.0274 (17)	0.0268 (19)	0.0277 (17)	0.0012 (14)	0.0107 (14)	−0.0047 (14)
C9B	0.0313 (18)	0.036 (2)	0.0269 (18)	−0.0041 (15)	0.0150 (15)	−0.0082 (15)
C10B	0.0272 (17)	0.036 (2)	0.0194 (16)	−0.0065 (15)	0.0076 (13)	0.0001 (14)
C11B	0.0252 (17)	0.0296 (19)	0.0281 (18)	0.0003 (14)	0.0053 (14)	0.0020 (14)
C12B	0.0241 (16)	0.0282 (19)	0.0245 (17)	−0.0012 (14)	0.0080 (13)	−0.0024 (13)
C13B	0.0196 (16)	0.0222 (18)	0.042 (2)	−0.0076 (13)	0.0103 (15)	0.0059 (15)
C14B	0.037 (2)	0.0258 (19)	0.0347 (19)	−0.0142 (15)	0.0149 (16)	−0.0081 (15)
C15B	0.0251 (18)	0.045 (2)	0.0240 (17)	−0.0177 (16)	0.0008 (14)	0.0019 (16)
C16B	0.0135 (15)	0.040 (2)	0.056 (2)	−0.0026 (15)	0.0083 (16)	0.0157 (18)
C17B	0.0267 (18)	0.038 (2)	0.049 (2)	−0.0041 (16)	0.0247 (17)	−0.0029 (17)
C18B	0.0298 (18)	0.039 (2)	0.0290 (18)	−0.0108 (16)	0.0152 (15)	0.0030 (15)
C19B	0.037 (2)	0.026 (2)	0.073 (3)	0.0047 (17)	0.019 (2)	0.010 (2)

### Chlorido{2-[(4-chlorophenyl)iminomethyl]pyridine-κ2N,N'}(η6-toluene)ruthenium(II) hexafluoridophosphate Geometric parameters (Å, º)

**Table:**

F7A—P1A	1.505 (8)	C19A—H19A	0.9800
F9A—P1A	1.536 (10)	C19A—H19B	0.9800
F10A—P1A	1.548 (10)	C19A—H19C	0.9800
Ru1A—Cl1A	2.3764 (8)	Ru1B—Cl1B	2.3821 (8)
Ru1A—N1A	2.081 (3)	Ru1B—N1B	2.081 (3)
Ru1A—N2A	2.090 (2)	Ru1B—N2B	2.083 (2)
Ru1A—C13A	2.199 (3)	Ru1B—C13B	2.222 (3)
Ru1A—C14A	2.194 (3)	Ru1B—C14B	2.187 (3)
Ru1A—C15A	2.191 (3)	Ru1B—C15B	2.184 (3)
Ru1A—C16A	2.199 (3)	Ru1B—C16B	2.165 (3)
Ru1A—C17A	2.184 (3)	Ru1B—C17B	2.183 (3)
Ru1A—C18A	2.182 (3)	Ru1B—C18B	2.203 (3)
Cl2A—C10A	1.738 (3)	Cl2B—C10B	1.746 (3)
P1A—F1A	1.596 (5)	P1B—F1B	1.576 (2)
P1A—F2A	1.561 (4)	P1B—F2B	1.587 (2)
P1A—F3A	1.583 (6)	P1B—F3B	1.592 (3)
P1A—F4A	1.601 (7)	P1B—F4B	1.595 (2)
P1A—F5A	1.548 (4)	P1B—F5B	1.587 (3)
P1A—F6A	1.595 (4)	P1B—F6B	1.580 (3)
P1A—F8A	1.545 (7)	N1B—C1B	1.338 (4)
P1A—F11A	1.572 (6)	N1B—C5B	1.356 (4)
P1A—F12A	1.551 (5)	N2B—C6B	1.279 (4)
N1A—C1A	1.340 (4)	N2B—C7B	1.436 (4)
N1A—C5A	1.357 (4)	C1B—H1B	0.9500
N2A—C6A	1.279 (4)	C1B—C2B	1.379 (5)
N2A—C7A	1.438 (4)	C2B—H2B	0.9500
C1A—H1A	0.9500	C2B—C3B	1.383 (5)
C1A—C2A	1.381 (5)	C3B—H3B	0.9500
C2A—H2A	0.9500	C3B—C4B	1.386 (5)
C2A—C3A	1.374 (5)	C4B—H4B	0.9500
C3A—H3A	0.9500	C4B—C5B	1.381 (4)
C3A—C4A	1.387 (5)	C5B—C6B	1.450 (4)
C4A—H4A	0.9500	C6B—H6B	0.9500
C4A—C5A	1.380 (5)	C7B—C8B	1.382 (4)
C5A—C6A	1.444 (4)	C7B—C12B	1.393 (4)
C6A—H6A	0.9500	C8B—H8B	0.9500
C7A—C8A	1.387 (4)	C8B—C9B	1.384 (4)
C7A—C12A	1.388 (4)	C9B—H9B	0.9500
C8A—H8A	0.9500	C9B—C10B	1.375 (5)
C8A—C9A	1.382 (4)	C10B—C11B	1.383 (5)
C9A—H9A	0.9500	C11B—H11B	0.9500
C9A—C10A	1.381 (5)	C11B—C12B	1.387 (4)
C10A—C11A	1.382 (5)	C12B—H12B	0.9500
C11A—H11A	0.9500	C13B—C14B	1.433 (5)
C11A—C12A	1.381 (4)	C13B—C18B	1.379 (5)
C12A—H12A	0.9500	C13B—C19B	1.491 (5)
C13A—C14A	1.427 (5)	C14B—H14B	0.9500
C13A—C18A	1.410 (4)	C14B—C15B	1.391 (5)
C13A—C19A	1.497 (5)	C15B—H15B	0.9500
C14A—H14A	0.9500	C15B—C16B	1.407 (5)
C14A—C15A	1.387 (5)	C16B—H16B	0.9500
C15A—H15A	0.9500	C16B—C17B	1.400 (5)
C15A—C16A	1.421 (5)	C17B—H17B	0.9500
C16A—H16A	0.9500	C17B—C18B	1.415 (5)
C16A—C17A	1.390 (5)	C18B—H18B	0.9500
C17A—H17A	0.9500	C19B—H19D	0.9800
C17A—C18A	1.413 (5)	C19B—H19E	0.9800
C18A—H18A	0.9500	C19B—H19F	0.9800
			
N1A—Ru1A—Cl1A	85.76 (7)	C17A—C18A—H18A	119.9
N1A—Ru1A—N2A	76.82 (10)	C13A—C19A—H19A	109.5
N1A—Ru1A—C13A	122.20 (11)	C13A—C19A—H19B	109.5
N1A—Ru1A—C14A	160.06 (12)	C13A—C19A—H19C	109.5
N1A—Ru1A—C15A	154.87 (12)	H19A—C19A—H19B	109.5
N1A—Ru1A—C16A	117.75 (12)	H19A—C19A—H19C	109.5
N1A—Ru1A—C17A	94.17 (12)	H19B—C19A—H19C	109.5
N1A—Ru1A—C18A	95.69 (11)	N1B—Ru1B—Cl1B	86.27 (7)
N2A—Ru1A—Cl1A	86.00 (7)	N1B—Ru1B—N2B	76.84 (10)
N2A—Ru1A—C13A	92.52 (11)	N1B—Ru1B—C13B	161.87 (12)
N2A—Ru1A—C14A	99.40 (11)	N1B—Ru1B—C14B	123.96 (12)
N2A—Ru1A—C15A	127.66 (12)	N1B—Ru1B—C15B	96.41 (12)
N2A—Ru1A—C16A	165.41 (12)	N1B—Ru1B—C16B	92.51 (12)
N2A—Ru1A—C17A	149.94 (12)	N1B—Ru1B—C17B	115.72 (12)
N2A—Ru1A—C18A	113.74 (11)	N1B—Ru1B—C18B	152.72 (12)
C13A—Ru1A—Cl1A	150.92 (9)	N2B—Ru1B—Cl1B	86.85 (7)
C14A—Ru1A—Cl1A	113.72 (9)	N2B—Ru1B—C13B	121.24 (11)
C14A—Ru1A—C13A	37.91 (12)	N2B—Ru1B—C14B	158.91 (12)
C14A—Ru1A—C16A	67.24 (13)	N2B—Ru1B—C15B	156.38 (13)
C15A—Ru1A—Cl1A	90.15 (9)	N2B—Ru1B—C16B	119.13 (13)
C15A—Ru1A—C13A	67.92 (13)	N2B—Ru1B—C17B	94.47 (12)
C15A—Ru1A—C14A	36.88 (13)	N2B—Ru1B—C18B	96.46 (12)
C15A—Ru1A—C16A	37.77 (13)	C13B—Ru1B—Cl1B	92.98 (9)
C16A—Ru1A—Cl1A	93.96 (9)	C14B—Ru1B—Cl1B	90.82 (10)
C16A—Ru1A—C13A	80.42 (12)	C14B—Ru1B—C13B	37.92 (12)
C17A—Ru1A—Cl1A	122.34 (10)	C14B—Ru1B—C18B	66.75 (13)
C17A—Ru1A—C13A	67.83 (12)	C15B—Ru1B—Cl1B	115.59 (11)
C17A—Ru1A—C14A	79.29 (12)	C15B—Ru1B—C13B	67.59 (13)
C17A—Ru1A—C15A	67.30 (13)	C15B—Ru1B—C14B	37.10 (13)
C17A—Ru1A—C16A	36.98 (13)	C15B—Ru1B—C18B	79.11 (13)
C18A—Ru1A—Cl1A	160.06 (9)	C16B—Ru1B—Cl1B	153.03 (12)
C18A—Ru1A—C13A	37.54 (12)	C16B—Ru1B—C13B	79.99 (13)
C18A—Ru1A—C14A	67.59 (12)	C16B—Ru1B—C14B	67.63 (14)
C18A—Ru1A—C15A	80.10 (13)	C16B—Ru1B—C15B	37.74 (14)
C18A—Ru1A—C16A	67.72 (13)	C16B—Ru1B—C17B	37.55 (14)
C18A—Ru1A—C17A	37.75 (12)	C16B—Ru1B—C18B	67.38 (13)
F7A—P1A—F9A	94.2 (9)	C17B—Ru1B—Cl1B	157.72 (10)
F7A—P1A—F10A	91.9 (9)	C17B—Ru1B—C13B	67.34 (13)
F7A—P1A—F8A	177.7 (6)	C17B—Ru1B—C14B	79.99 (14)
F7A—P1A—F11A	89.9 (5)	C17B—Ru1B—C15B	67.77 (14)
F7A—P1A—F12A	93.8 (5)	C17B—Ru1B—C18B	37.63 (13)
F9A—P1A—F10A	173.6 (9)	C18B—Ru1B—Cl1B	120.10 (10)
F9A—P1A—F8A	84.3 (9)	C18B—Ru1B—C13B	36.31 (13)
F9A—P1A—F11A	87.8 (5)	F1B—P1B—F2B	179.44 (17)
F9A—P1A—F12A	90.4 (5)	F1B—P1B—F3B	90.34 (14)
F10A—P1A—F11A	94.0 (7)	F1B—P1B—F4B	89.45 (14)
F10A—P1A—F12A	87.4 (6)	F1B—P1B—F5B	89.99 (16)
F1A—P1A—F4A	84.7 (4)	F1B—P1B—F6B	91.13 (17)
F2A—P1A—F1A	177.1 (4)	F2B—P1B—F3B	90.11 (15)
F2A—P1A—F3A	92.1 (4)	F2B—P1B—F4B	90.10 (14)
F2A—P1A—F4A	93.7 (4)	F2B—P1B—F5B	90.35 (16)
F2A—P1A—F6A	90.9 (3)	F3B—P1B—F4B	179.79 (15)
F3A—P1A—F1A	89.3 (4)	F5B—P1B—F3B	89.81 (15)
F3A—P1A—F4A	173.5 (4)	F5B—P1B—F4B	90.13 (16)
F3A—P1A—F6A	87.5 (3)	F6B—P1B—F2B	88.53 (15)
F5A—P1A—F1A	91.8 (3)	F6B—P1B—F3B	89.68 (16)
F5A—P1A—F2A	90.6 (4)	F6B—P1B—F4B	90.38 (16)
F5A—P1A—F3A	93.6 (3)	F6B—P1B—F5B	178.77 (17)
F5A—P1A—F4A	89.2 (3)	C1B—N1B—Ru1B	126.3 (2)
F5A—P1A—F6A	178.1 (3)	C1B—N1B—C5B	118.3 (3)
F6A—P1A—F1A	86.6 (3)	C5B—N1B—Ru1B	115.4 (2)
F6A—P1A—F4A	89.5 (3)	C6B—N2B—Ru1B	116.3 (2)
F8A—P1A—F10A	89.7 (9)	C6B—N2B—C7B	119.6 (3)
F8A—P1A—F11A	88.4 (5)	C7B—N2B—Ru1B	124.1 (2)
F8A—P1A—F12A	87.9 (5)	N1B—C1B—H1B	118.9
F12A—P1A—F11A	176.0 (4)	N1B—C1B—C2B	122.3 (3)
C1A—N1A—Ru1A	126.4 (2)	C2B—C1B—H1B	118.9
C1A—N1A—C5A	118.2 (3)	C1B—C2B—H2B	120.2
C5A—N1A—Ru1A	115.4 (2)	C1B—C2B—C3B	119.6 (3)
C6A—N2A—Ru1A	116.0 (2)	C3B—C2B—H2B	120.2
C6A—N2A—C7A	118.4 (3)	C2B—C3B—H3B	120.7
C7A—N2A—Ru1A	125.6 (2)	C2B—C3B—C4B	118.5 (3)
N1A—C1A—H1A	119.1	C4B—C3B—H3B	120.7
N1A—C1A—C2A	121.8 (3)	C3B—C4B—H4B	120.5
C2A—C1A—H1A	119.1	C5B—C4B—C3B	119.1 (3)
C1A—C2A—H2A	119.9	C5B—C4B—H4B	120.5
C3A—C2A—C1A	120.2 (3)	N1B—C5B—C4B	122.1 (3)
C3A—C2A—H2A	119.9	N1B—C5B—C6B	113.7 (3)
C2A—C3A—H3A	120.8	C4B—C5B—C6B	124.2 (3)
C2A—C3A—C4A	118.3 (3)	N2B—C6B—C5B	117.5 (3)
C4A—C3A—H3A	120.8	N2B—C6B—H6B	121.2
C3A—C4A—H4A	120.4	C5B—C6B—H6B	121.2
C5A—C4A—C3A	119.1 (3)	C8B—C7B—N2B	120.7 (3)
C5A—C4A—H4A	120.4	C8B—C7B—C12B	121.3 (3)
N1A—C5A—C4A	122.2 (3)	C12B—C7B—N2B	118.0 (3)
N1A—C5A—C6A	113.8 (3)	C7B—C8B—H8B	120.3
C4A—C5A—C6A	124.0 (3)	C7B—C8B—C9B	119.5 (3)
N2A—C6A—C5A	117.9 (3)	C9B—C8B—H8B	120.3
N2A—C6A—H6A	121.1	C8B—C9B—H9B	120.5
C5A—C6A—H6A	121.1	C10B—C9B—C8B	119.0 (3)
C8A—C7A—N2A	119.4 (3)	C10B—C9B—H9B	120.5
C8A—C7A—C12A	120.9 (3)	C9B—C10B—Cl2B	118.9 (3)
C12A—C7A—N2A	119.7 (3)	C9B—C10B—C11B	122.2 (3)
C7A—C8A—H8A	120.3	C11B—C10B—Cl2B	118.9 (3)
C9A—C8A—C7A	119.4 (3)	C10B—C11B—H11B	120.6
C9A—C8A—H8A	120.3	C10B—C11B—C12B	118.9 (3)
C8A—C9A—H9A	120.3	C12B—C11B—H11B	120.6
C10A—C9A—C8A	119.3 (3)	C7B—C12B—H12B	120.5
C10A—C9A—H9A	120.3	C11B—C12B—C7B	119.1 (3)
C9A—C10A—Cl2A	119.5 (3)	C11B—C12B—H12B	120.5
C9A—C10A—C11A	121.7 (3)	C14B—C13B—Ru1B	69.71 (19)
C11A—C10A—Cl2A	118.8 (3)	C14B—C13B—C19B	119.9 (3)
C10A—C11A—H11A	120.5	C18B—C13B—Ru1B	71.1 (2)
C12A—C11A—C10A	119.0 (3)	C18B—C13B—C14B	118.4 (3)
C12A—C11A—H11A	120.5	C18B—C13B—C19B	121.7 (3)
C7A—C12A—H12A	120.2	C19B—C13B—Ru1B	129.5 (2)
C11A—C12A—C7A	119.7 (3)	Ru1B—C14B—H14B	128.9
C11A—C12A—H12A	120.2	C13B—C14B—Ru1B	72.37 (18)
C14A—C13A—Ru1A	70.83 (18)	C13B—C14B—H14B	119.8
C14A—C13A—C19A	121.3 (3)	C15B—C14B—Ru1B	71.3 (2)
C18A—C13A—Ru1A	70.58 (18)	C15B—C14B—C13B	120.5 (3)
C18A—C13A—C14A	118.2 (3)	C15B—C14B—H14B	119.8
C18A—C13A—C19A	120.5 (3)	Ru1B—C15B—H15B	130.7
C19A—C13A—Ru1A	128.7 (2)	C14B—C15B—Ru1B	71.56 (18)
Ru1A—C14A—H14A	130.7	C14B—C15B—H15B	120.0
C13A—C14A—Ru1A	71.26 (18)	C14B—C15B—C16B	120.0 (3)
C13A—C14A—H14A	119.4	C16B—C15B—Ru1B	70.39 (19)
C15A—C14A—Ru1A	71.5 (2)	C16B—C15B—H15B	120.0
C15A—C14A—C13A	121.3 (3)	Ru1B—C16B—H16B	128.7
C15A—C14A—H14A	119.4	C15B—C16B—Ru1B	71.88 (19)
Ru1A—C15A—H15A	129.3	C15B—C16B—H16B	119.8
C14A—C15A—Ru1A	71.66 (19)	C17B—C16B—Ru1B	71.92 (19)
C14A—C15A—H15A	120.0	C17B—C16B—C15B	120.4 (3)
C14A—C15A—C16A	120.0 (3)	C17B—C16B—H16B	119.8
C16A—C15A—Ru1A	71.4 (2)	Ru1B—C17B—H17B	129.2
C16A—C15A—H15A	120.0	C16B—C17B—Ru1B	70.5 (2)
Ru1A—C16A—H16A	130.4	C16B—C17B—H17B	120.6
C15A—C16A—Ru1A	70.80 (18)	C16B—C17B—C18B	118.9 (3)
C15A—C16A—H16A	120.4	C18B—C17B—Ru1B	71.95 (19)
C17A—C16A—Ru1A	70.90 (19)	C18B—C17B—H17B	120.6
C17A—C16A—C15A	119.2 (3)	Ru1B—C18B—H18B	130.8
C17A—C16A—H16A	120.4	C13B—C18B—Ru1B	72.62 (19)
Ru1A—C17A—H17A	130.0	C13B—C18B—C17B	121.9 (3)
C16A—C17A—Ru1A	72.12 (18)	C13B—C18B—H18B	119.0
C16A—C17A—H17A	119.4	C17B—C18B—Ru1B	70.4 (2)
C16A—C17A—C18A	121.2 (3)	C17B—C18B—H18B	119.0
C18A—C17A—Ru1A	71.08 (17)	C13B—C19B—H19D	109.5
C18A—C17A—H17A	119.4	C13B—C19B—H19E	109.5
Ru1A—C18A—H18A	129.4	C13B—C19B—H19F	109.5
C13A—C18A—Ru1A	71.88 (18)	H19D—C19B—H19E	109.5
C13A—C18A—C17A	120.1 (3)	H19D—C19B—H19F	109.5
C13A—C18A—H18A	119.9	H19E—C19B—H19F	109.5
C17A—C18A—Ru1A	71.17 (18)		
			
Ru1A—N1A—C1A—C2A	−177.9 (2)	Ru1B—N1B—C1B—C2B	−176.1 (2)
Ru1A—N1A—C5A—C4A	176.9 (3)	Ru1B—N1B—C5B—C4B	175.8 (3)
Ru1A—N1A—C5A—C6A	−3.7 (4)	Ru1B—N1B—C5B—C6B	−5.4 (3)
Ru1A—N2A—C6A—C5A	1.5 (4)	Ru1B—N2B—C6B—C5B	2.0 (4)
Ru1A—N2A—C7A—C8A	−43.7 (4)	Ru1B—N2B—C7B—C8B	135.9 (3)
Ru1A—N2A—C7A—C12A	135.1 (3)	Ru1B—N2B—C7B—C12B	−43.0 (4)
Ru1A—C13A—C14A—C15A	53.2 (3)	Ru1B—C13B—C14B—C15B	54.8 (3)
Ru1A—C13A—C18A—C17A	−54.4 (3)	Ru1B—C13B—C18B—C17B	−52.5 (3)
Ru1A—C14A—C15A—C16A	54.6 (3)	Ru1B—C14B—C15B—C16B	53.1 (3)
Ru1A—C15A—C16A—C17A	53.8 (3)	Ru1B—C15B—C16B—C17B	55.2 (3)
Ru1A—C16A—C17A—C18A	53.6 (3)	Ru1B—C16B—C17B—C18B	55.2 (3)
Ru1A—C17A—C18A—C13A	54.7 (3)	Ru1B—C17B—C18B—C13B	53.5 (3)
Cl2A—C10A—C11A—C12A	−177.9 (2)	Cl2B—C10B—C11B—C12B	−179.9 (3)
N1A—C1A—C2A—C3A	−0.4 (5)	N1B—C1B—C2B—C3B	0.3 (5)
N1A—C5A—C6A—N2A	1.5 (4)	N1B—C5B—C6B—N2B	2.2 (4)
N2A—C7A—C8A—C9A	−178.9 (3)	N2B—C7B—C8B—C9B	179.9 (3)
N2A—C7A—C12A—C11A	179.2 (3)	N2B—C7B—C12B—C11B	179.9 (3)
C1A—N1A—C5A—C4A	−4.1 (5)	C1B—N1B—C5B—C4B	−3.1 (5)
C1A—N1A—C5A—C6A	175.3 (3)	C1B—N1B—C5B—C6B	175.7 (3)
C1A—C2A—C3A—C4A	−1.5 (5)	C1B—C2B—C3B—C4B	−2.9 (5)
C2A—C3A—C4A—C5A	0.7 (5)	C2B—C3B—C4B—C5B	2.5 (5)
C3A—C4A—C5A—N1A	2.2 (5)	C3B—C4B—C5B—N1B	0.5 (5)
C3A—C4A—C5A—C6A	−177.1 (3)	C3B—C4B—C5B—C6B	−178.2 (3)
C4A—C5A—C6A—N2A	−179.2 (3)	C4B—C5B—C6B—N2B	−178.9 (3)
C5A—N1A—C1A—C2A	3.2 (5)	C5B—N1B—C1B—C2B	2.7 (5)
C6A—N2A—C7A—C8A	137.3 (3)	C6B—N2B—C7B—C8B	−43.3 (4)
C6A—N2A—C7A—C12A	−43.9 (4)	C6B—N2B—C7B—C12B	137.8 (3)
C7A—N2A—C6A—C5A	−179.4 (3)	C7B—N2B—C6B—C5B	−178.7 (3)
C7A—C8A—C9A—C10A	−0.8 (5)	C7B—C8B—C9B—C10B	0.4 (5)
C8A—C7A—C12A—C11A	−2.0 (5)	C8B—C7B—C12B—C11B	1.0 (5)
C8A—C9A—C10A—Cl2A	178.2 (3)	C8B—C9B—C10B—Cl2B	179.7 (3)
C8A—C9A—C10A—C11A	−1.0 (5)	C8B—C9B—C10B—C11B	0.7 (5)
C9A—C10A—C11A—C12A	1.3 (5)	C9B—C10B—C11B—C12B	−0.9 (5)
C10A—C11A—C12A—C7A	0.2 (5)	C10B—C11B—C12B—C7B	0.0 (5)
C12A—C7A—C8A—C9A	2.3 (5)	C12B—C7B—C8B—C9B	−1.2 (5)
C13A—C14A—C15A—Ru1A	−53.1 (3)	C13B—C14B—C15B—Ru1B	−55.3 (3)
C13A—C14A—C15A—C16A	1.5 (5)	C13B—C14B—C15B—C16B	−2.2 (5)
C14A—C13A—C18A—Ru1A	54.3 (2)	C14B—C13B—C18B—Ru1B	53.0 (3)
C14A—C13A—C18A—C17A	−0.1 (4)	C14B—C13B—C18B—C17B	0.5 (5)
C14A—C15A—C16A—Ru1A	−54.7 (3)	C14B—C15B—C16B—Ru1B	−53.6 (3)
C14A—C15A—C16A—C17A	−0.9 (5)	C14B—C15B—C16B—C17B	1.6 (5)
C15A—C16A—C17A—Ru1A	−53.7 (3)	C15B—C16B—C17B—Ru1B	−55.2 (3)
C15A—C16A—C17A—C18A	−0.1 (5)	C15B—C16B—C17B—C18B	0.0 (5)
C16A—C17A—C18A—Ru1A	−54.1 (3)	C16B—C17B—C18B—Ru1B	−54.5 (3)
C16A—C17A—C18A—C13A	0.7 (5)	C16B—C17B—C18B—C13B	−1.1 (5)
C18A—C13A—C14A—Ru1A	−54.2 (3)	C18B—C13B—C14B—Ru1B	−53.7 (3)
C18A—C13A—C14A—C15A	−1.0 (5)	C18B—C13B—C14B—C15B	1.1 (5)
C19A—C13A—C14A—Ru1A	124.3 (3)	C19B—C13B—C14B—Ru1B	124.7 (3)
C19A—C13A—C14A—C15A	177.5 (3)	C19B—C13B—C14B—C15B	179.5 (3)
C19A—C13A—C18A—Ru1A	−124.2 (3)	C19B—C13B—C18B—Ru1B	−125.3 (3)
C19A—C13A—C18A—C17A	−178.6 (3)	C19B—C13B—C18B—C17B	−177.8 (3)

### Chlorido{2-[(4-chlorophenyl)iminomethyl]pyridine-κ2N,N'}(η6-toluene)ruthenium(II) hexafluoridophosphate Hydrogen-bond geometry (Å, º)

**Table:**

*D*—H···*A*	*D*—H	H···*A*	*D*···*A*	*D*—H···*A*
C1*A*—H1*A*···F7*A*^i^	0.95	2.33	3.242 (11)	160
C3*A*—H3*A*···F2*A*^ii^	0.95	2.43	3.261 (8)	147
C19*A*—H19*A*···F11*A*^iii^	0.98	2.38	3.243 (9)	146
C15*B*—H15*B*···F4*B*^iv^	0.95	2.35	3.292 (4)	172

Symmetry codes: (i) *x*, −*y*+3/2, *z*−1/2; (ii) −*x*, −*y*+1, −*z*; (iii) −*x*, *y*+1/2, −*z*+1/2; (iv) *x*, −*y*+1/2, *z*+1/2.
